# Optimal Array Design and Directive Sensors for Guided Waves DoA Estimation

**DOI:** 10.3390/s22030780

**Published:** 2022-01-20

**Authors:** Marco Dibiase, Masoud Mohammadgholiha, Luca De Marchi

**Affiliations:** 1 Department of Computer Science and Engineering, University of Bologna, 40136 Bologna, Italy; marco.dibiase3@unibo.it; 2 Department of Electrical, Electronic, and Information Engineering “Guglielmo Marconi”, University of Bologna, 40136 Bologna, Italy; m.mohammadgholiha@unibo.it

**Keywords:** direction of arrival, structural health monitoring, array design, doa efficient estimator, directive piezoelectric sensor, guided waves, cramér-rao matrix bound, bayesian criterion

## Abstract

The estimation of Direction of Arrival (DoA) of guided ultrasonic waves is an important task in many Structural Health Monitoring (SHM) applications. The aim is to locate sources of elastic waves which can be generated by impacts or defects in the inspected structures. In this paper, the array geometry and the shape of the piezo-sensors are designed to optimize the DoA estimation on a pre-defined angular sector, from acquisitions affected by noise and interference. In the proposed approach, the DoA of a wave generated by a single source is considered as a random variable that is uniformly distributed in a given range. The wave velocity is assumed to be unknown and the DoA estimation is performed by measuring the Differences in Time of Arrival (DToAs) of wavefronts impinging on the sensors. The optimization procedure of sensors positioning is based on the computation of the DoA and wave velocity parameters Cramér-Rao Matrix Bound (CRMB) with a Bayesian approach. An efficient DoA estimator is found based on the DToAs Gauss-Markov estimator for a three sensors array. Moreover, a novel directive sensor for guided waves is introduced to cancel out undesired Acoustic Sources impinging from DoAs out of the given angles range. Numerical results show the capability to filter directional interference of the novel sensor and a considerably improved DoA estimation performance provided by the optimized sensor cluster in the pre-defined angular sector, as compared to conventional approaches.

## 1. Introduction

Acoustic source (AS) localization from the measurements of passive sensors is a widely-investigated problem in structural health monitoring (SHM) [1,2]. An AS could consist of undesired impacts on the monitored structure. Alternatively, Acoustic Emissions (AE) can be generated by the growth of defects, such as cracks and corrosion. Acoustic sources in plate-like structures generate Lamb waves [3], i.e., guided stress waves (GW). The detection by means of piezoelectric sensors and the subsequent analysis of AS signals allow to infer whether an impact has occurred and to localize it. Although several methods for AS localization were proposed and validated at laboratory scale, these approaches rarely satisfy the following stringent constraints which are posed by field applications:

1. Minimal monitoring system weight, including cabling and circuitry;
2. Minimal power consumption, to be compatible with wireless systems. This means reducing the computational cost for the signals local processing and/or the amount of data to be wirelessly transferred;

Among all methods of AS localization, three main typologies can be identified:

1. Inverse methods [4,5];
2. Hyperbolic positioning [6];
3. Methods which use Directions of Arrival (DoAs) estimation [7,8,9];

The first typology requires very accurate modeling of ultrasonic propagation. It can be performed by knowing previously the structural parameters or by measuring them. However, the main limitations of inverse methods, are that they require a high computational cost and, consequently, are incompatible with lightweight and low-power embedded systems. The second typology estimates the AS location as the intersection point of geometric hyperbola derived by the time delays between sensors. The hyperbolic positioning methods require a large number of distant and well-synchronized sensors in order to reduce the estimation uncertainty. Unfortunately, accurate synchronization is difficult to implement in a wireless system. For these reasons, the methods of the third typology, which estimate DoAs by using clusters of closely located sensors, are often the only feasible approach to AS localization. Indeed, these methods have a computational cost compatible with embedded systems and do not require synchronization between distant sensors.

Different strategies which use one or more clusters for the AS estimation have been studied and tested [7,8,9]. Among these, the first two [7,8] use just one cluster to locate an AS. Nevertheless, in order to estimate the AS distance, they require to detect two modes (typically the A0 and S0 modes) and compute their time difference on the sensors. Conversely, the technique proposed by Kundu [9] is suitable even when one only wave mode is detected. This is the case of AS generated by an impact, when, typically, the Lamb waves A0 fundamental mode has a much higher amplitude w.r.t. other modes. Such technique uses–at least–two closely spaced sensors clusters placed apart to locate sources both in isotropic and slightly anisotropic plates. The DoA estimation performance of a simple cluster of three circular sensors placed on the vertices of an isosceles right triangle, with unknown wave velocity, was investigated in [10,11]. The DoA estimation is performed by means of the Differences in Time of Arrival (DToAs) estimations, via simple Cross-Correlation (CC) procedures. In this work, this cluster will be referred to as *Standard Cluster* (SC). In [12,13], it was shown that it is possible to use multiple clusters to estimate the AS localization even in the case of heavily anisotropic structures (i.e., characterized by elliptical or rhomboidal wavefronts).

Although the SC using, for AS location, was well-validated at laboratory scale, it is well-known that in realistic field deployment, several problems arise due to noise and directional interference. Typical noisy physical sources are given by: structural vibration (e.g., due to turbulence on an aircraft), scattered wavefield, noisy acquisition channels, or noisy electronic devices. Whereas the directive interference is due to the waves reverberation within bounded structures. In particular, we can distinguish coherent interference w.r.t the signal to be detected and incoherent signals. The first ones are due to edge-reflections of the impact/defect AE to be detected. The second ones are due to reflections produced by different impacts or acoustic events.

Several studies have been already presented to address the noise and the reverberation issues in DoA estimation and AS localization. In [14], Oktel and Moses proposed a sensors cluster design procedure to increase the DoA estimation performance in presence of noise. It was based on the Bayesian approach (or *global*) of the Cramér Rao bound (CRB), which depends on the sensors positioning and defines the lower bound of any unbiased estimator. However, in this work, the wave velocity is supposed to be known. Conversely, in many applications, such an assumption is not verified and results in a loss in accuracy.

Regarding the directional interference issue, several estimation methods were proposed to filter or distinguish the undesired corruption from the useful signal. In particular, among the DoA estimators based on the DToAs estimations, the Generalized CC (GCC) procedure with phase transform (GCC-PHAT), has been shown to be a suitable alternative in reverberant scenarios [15,16]. However, its performance can only be considered optimal for a high signal-to-noise ratio (SNR) (as shown in [17]). As a consequence, GCC-PHAT is not suited for the applications considered in this work.

A different DoA estimation method, the Multiple Signal Classification (MUSIC) [18], is able to estimate up to N-1 DoAs due to different sources with N-sensor arrays. Originally designed to estimate the number and DoAs of uncorrelated signals, modified versions [19,20] have been proposed to estimate also the DoAs of coherent signals for the multipath environment. However, with a simple 3-sensor cluster, just 2 coherent signals can be detected. This means that other directional interferences may cause wrong estimations. A more robust MUSIC algorithm for reverberant scenarios is proposed in [21]. MUSIC algorithms are also limited by the assumption of accurate knowledge or estimation of wave velocity. Therefore, an additional iterative wave velocity estimation procedure is needed (as shown in [22]), which increases consistently the computational cost.

This work provides original solutions to tackle the detrimental effects both of noise and of directional interference. The first problem is addressed by means of a novel strategy for sensor cluster design. Unlike [14], we considered the *velocity to be unknown*. *In our approach, the cluster design procedure* is based on the computation of the CRB of DoA in case of the unknown velocity of propagation (CRB${}_{u-v}$), and on the usage of its Bayesian average (assuming that the DoA is a random variable with a known probability distribution) as a cost function for the optimal design. This allows us to minimize both the DoA lower bound and the DoA accuracy loss due to the unknown wave velocity.

The second problem, i.e., the negative impact of directional interference, is tackled by means of *novel directive piezo-sensors, suitable for guided propagation structures*. The shaping of piezoelectric transducers has already been used as a powerful means to detect DoA in non-reverberant contexts [23,24]. Here, we show how the shaping can be used to filter out all directional interference, either coherent or incoherent w.r.t. the useful wave signal. It is worth noting that the usage of these transducers is beneficial whenever a limited angular sector has to be monitored, without regard to the number of sensors and the adopted DoA estimator.

The design procedure for the directive transducer proposed in this paper draws inspiration from the work of Senesi and Ruzzene [25] which showed how to relate the transducer shape to its directivity. However, the transducers considered in [25] are characterized by symmetric beam patterns which are unsuited to distinguish sources related to opposite directions. To achieve this capability, a *novel complex (i.e., multi-phase) transducer* has to be implemented [26]. The proposed *Directive Complex sensor* (DCS) consists of five piezoelectric patches and allows to suppress the lobes out of a 90° monitoring sector. The experimental validation of the piezo-patches shaping design is beyond the scope of this paper. Nevertheless, future developments are aimed to realize the proposed DCS, by exploiting the available manufacturing techniques. For example, a laser-cut can be used to shape piezo-electrodes on the upper surface of metalized PVDF (polyvinylidene fluoride) sheets, as proposed in [27]. Alternatively, a metallic printing technique can be used on PVDF films to obtain the desired shape patches [28].

The details of the cluster design strategy, the adopted DoA estimator, the novel directional transducer concept and, finally, the numerical validations are thoroughly illustrated in the following sections.

## 2. System Model and Cramér-Rao Matrix

Let us assume that the sensors array consists of three identical sensors: P1, P2, and P3. The sensors are located at $r_{i}={\left[x_{i},y_{i}\right]}^{T}$ for i∈[1,2,3]. Following [6,29], we adopt a model with a single co-planar far-field source which generates the wavefield impinging the 3 sensors array. The signal at the *i*th sensor is $s(t-d_{i})$, where s(t) is the signal at a reference point near the array and $d_{i}$ is the delay at the *i*th sensor w.r.t. the reference point. Without loss of generality, the reference point is assumed to be coincident with the location of the first element in the array, P1, so that $d_{1}=0$. We assume also that the sensors are near enough so that the amplitude gradient across the array and the effect of wave dispersion are negligible. The output signal of the *i*th sensor can be expressed as:

(1) $$x_{i}\left(t\right)=s\left(t-d_{i}\right)+n_{i}\left(t\right)$$

where $n_{i}\left(t\right)$ is the additive sensor noise at *i*th sensor. In order to estimate the DoA of wavefront, we first estimate the vector of DToAs, $d={\left[d_{2},d_{3}\right]}^{T}$. In the discrete Fourier domain, the 3 × 1 measurements vector at *k*th frequency $\omega_{k}$, is given by:

(2) $$x\left(\omega_{k}\right)=a_{\theta }\left(\omega_{k}\right)s\left(\omega_{k}\right)+n\left(\omega_{k}\right)$$

where $a_{\theta }\left(\omega_{k}\right)$ is the steering vector, defined as:

(3) $$a_{\theta }\left(\omega_{k}\right)={\left[1,e^{j\omega_{k}d_{2}\left(\theta \right)},e^{j\omega_{k}d_{3}\left(\theta \right)}\right]}^{T}$$

where $d_{i}\left(\theta \right)=\left(u^{T}\left(\theta \right)\cdot r_{i}-u^{T}\left(\theta \right)\cdot r_{1}\right)/v$ is the DToA between the *i*th sensor and the reference sensor P1, *v* is the wave velocity and $u\left(\theta \right)={\left[\cos(\theta ),\sin(\theta )\right]}^{T}$ is the unit vector pointing toward the signal source. The followings hypotheses are formulated:

1. The noises are stationary Gaussian processes with zero mean. The signal is a Gaussian process with zero means, approximately stationary. The last hypothesis assumes that, for closely spaced sensors, the dispersion effect can be neglected;
2. The signal and noises are mutually uncorrelated and uncorrelated between themselves;

Under the previous hypotheses, Hahn and Tretter in [29], derived the Cramér-Rao Matrix Bound (CRMB), Q, for the delays. We suppose that an optimal estimator is used to estimate the DToAs, such as the Maximum Likelihood (ML) estimator proposed in [29], therefore the covariance matrix is equal to the CRMB. Moreover, we suppose that noises have identical covariance matrix. This means that the noises have identical spectrum and in case of white noises, they have the same noise level. When the noises have same spectrum, the covariance matrix Q assumes the following simple form:

(4) $$Q=\sigma_{d_{i}}^{2}\left[\begin{array}{cc} 1 & 1/2\\ 1/2 & 1 \end{array}\right]$$

where the variance of time-delays, $\sigma_{d_{i}}^{2}$, is given by:

(5) $$\sigma_{d_{i}}^{2}=\frac{2}{3}\frac{2\pi }{T_{S}\int_{0}^{B_{S}}2\omega^{2}\frac{S^{2}/N^{2}}{1+3(S/N)}d\omega }$$

where S(ω) and N(ω) are the power spectra of signal and noise, $B_{S}$ is the signal band and $T_{S}$ is the signal time duration. Thanks to the asymptotically Gaussian property of a ML estimator, the conditional probability density function of d is:

(6) $$f\left(d;\theta \right)=\frac{1}{{\left(2\pi \right)}^{2}\sqrt{\det Q}}\exp\left\{-\frac{1}{2}{\left(d-\frac{r}{v}\right)}^{T}Q^{-1}\left(d-\frac{r}{v}\right)\right\}$$

with $r={\left[r_{2}\left(\theta \right),r_{3}\left(\theta \right)\right]}^{T}$ the vector of Differences in Distance of Arrival (DDoAs) of the wavefront between the sensors and the reference sensor P1. Note that $r_{i}\left(\theta \right)$ (i=2,3) are so that the “true” values of $d_{i}\left(\theta \right)$ are $d_{i}\left(\theta \right)=r_{i}\left(\theta \right)/v={\left(u^{T}\left(\theta \right)\cdot r_{i}-u^{T}\left(\theta \right)\cdot r_{1}\right)}_{i}/v$. Let’s assume that the wave velocity is unknown. So, the unknown parameters are θ and *v*. In [30], Malagò and Pistone provided the Fisher information Matrix (FIM) for a Gaussian distribution when the vector of the means μ and the covariance matrix Q are both functions of a set of parameters $\gamma ={\left(\gamma_{1},\gamma_{2},...,\gamma_{K}\right)}^{T}$. The expression, specialized in our case (with $\gamma_{1}=\theta$ and $\gamma_{2}=v$), is the following:

(7) $$I_{m,n}\left(\gamma \right)=\frac{\partial \left[r_{2}\left(\theta \right)/v,r_{3}\left(\theta \right)/v\right]}{\partial \gamma_{m}}Q^{-1}\frac{\partial \left[\begin{array}{c} r_{2}\left(\theta \right)/v\\ r_{3}\left(\theta \right)/v \end{array}\right]}{\partial \gamma_{m}}$$

with $Q^{-1}$, from (4) is given by:

(8) $$Q^{-1}=\frac{4}{3\sigma_{d_{i}}^{2}}\left[\begin{array}{cc} 1 & -1/2\\ -1/2 & 1 \end{array}\right]$$

The inverse of the FIM provided in Equation (7) is the sought CRMB for unknown parameters θ and *v*, which defines a lower bound of the covariance matrix for any unbiased estimator of two parameters.

## 3. Cost Function and Array Geometry Design

The D-criterion (see [31]) uses the determinant of CRMB (equal to the inverse of FIM determinant, often called generalized covariance bound) as a cost function to be minimized to obtain the optimal design. However, in the current application domain, only the DoA estimation performance has to be optimized. Therefore, the following function has been considered:

(9) $$\frac{\det(\mathrm{FIM})}{I_{22}}=I_{11}-\frac{I_{12}I_{21}}{I_{22}}$$

It consists of two terms: the first one is related only to the Fisher Information (FI) on θ (when *v* is known) and the second one is related to information on θ and *v*, when they are simultaneously estimated, divided by the FI on *v* (when θ is known). Finally, adopting a Bayesian (or *global*) approach, similarly to [14], the following CRB${}_{v-u}$ cost function is defined:

(10) $$\begin{array}{c} J_{C}\left(r\right)=E\left[I_{22}\det(\mathrm{CRMB})\right]=E\left[\frac{I_{22}}{\det(\mathrm{FIM})}\right]\\ =\frac{1}{2\theta_{0}}\int_{-\theta_{0}}^{\theta_{0}}\frac{I_{22}\left(r\left(\theta \right)\right)}{\det(\mathrm{FIM}(r(\theta )))}f\left(\theta \right)d\theta \end{array}$$

where f(θ) is the probability density function (pdf) of θ, thought as random variable and $\left[-\theta_{0},\theta_{0}\right]$ is its domain, supposed compact. The column vector r(θ) is function of sensors locations coordinates as:

(11) $$r\left(\theta \right)=\left[\begin{array}{c} \left(x_{2}-x_{1}\right)\cos\left(\theta \right)+\left(y_{2}-y_{1}\right)\sin\left(\theta \right)\\ \left(x_{3}-x_{1}\right)\cos\left(\theta \right)+\left(y_{3}-y_{1}\right)\sin\left(\theta \right) \end{array}\right]$$

We define the CRB${}_{u-v}$-optimal array $r_{C}$ as the one whose elements location is given by:

(12) $$\begin{array}{c} r_{C}=\arg\min J_{C}\left(r\right)\\ \mathrm{with}\;\left\{\begin{array}{c} \sqrt{x_{1}^{2}+y_{1}^{2}}\leq d\;\\ \sqrt{x_{2}^{2}+y_{2}^{2}}\leq d\\ \sqrt{x_{3}^{2}+y_{3}^{2}}\leq d \end{array}\right. \end{array}$$

where *d* is the radius of the circular domain where the sensor elements are constrained to lie in.

The general problem statement can be specified to the case of uniform pdf in a 90° sector, i.e., $\left[-\pi /4,\pi /4\right]$. Computing the terms $I_{m,n}$ of (7), the cost function (10) is:

(13) $$J_{C}\left(r\right)=\sigma_{d_{i}}^{2}\frac{2v^{2}}{\pi }\int_{-\pi /4}^{\pi /4}\frac{r_{2}^{2}\left(\theta \right)+r_{3}^{2}\left(\theta \right)-r_{2}\left(\theta \right)r_{3}\left(\theta \right)}{{\left({r_{2}}^{\prime }\left(\theta \right)r_{3}\left(\theta \right)-r_{2}\left(\theta \right){r_{3}}^{\prime }\left(\theta \right)\right)}^{2}}d\theta$$

**Figure 1:**
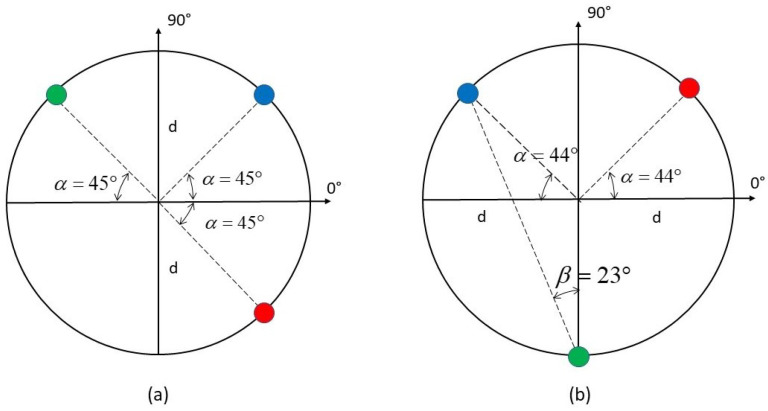
(**a**) Standard Cluster of three sensors (SC). (**b**) Designed Cluster (DC) of three sensors, optimized for DoA estimation in [−45°,45°] sector with unknown velocity *v*.

Its minimization is obtained with a symmetric configuration with respect to 90°-axis (i.e., Y-axis), and a half-opening angle β equal to 23° (see Figure 1b). Conversely, when the wave velocity is known, the optimized array would be symmetric w.r.t the X-axis [14]. This particular configuration is due to the minimization of the DoA accuracy loss due to the unknown wave velocity [32], via Equation (13). The obtained 3-sensor cluster will be referred to as Designed Cluster (DC) while the configuration of Figure 1a, which is related to the cluster proposed by Kundu et al. in [10,11], will be referred to as Standard Cluster (SC) in this paper.

Finally, it is worth noting that the previous design procedure of the optimal sensor positioning is still valid for a generic number *M* of sensors, with an appropriate covariance matrix (4).

## 4. A DoA Efficient Estimator

As anticipated, an efficient time delays estimator has to be used in order to match the DToAs covariance matrix with the CRMB (4). The Maximum Likelihood (ML) DToAs estimator, asymptotically efficient, was proposed by Hahn and Tretter in [29]. The technique consists of measuring the DToAs for all possible sensors pairs by Generalized Cross Correlation (GCC) and then calculating the Gauss-Markov (GM) (weighted) estimate of the DToAs with respect to the first sensor. The GCC procedure consists of computing the Cross-Correlation between the acquired signals, filtered first by an appropriate filter. The Optimal Filtering to attain the time delays CRMB is defined by:

(14) $${\left|F_{OPT(\omega )}\right|}^{2}=\frac{S\left(\omega \right)/N^{2}\left(\omega \right)}{1+M(S(\omega )/N(\omega ))}$$

where *M* is the number of the sensor, whereas *S* and *N* are the power spectra of respectively no-noisy signal and noise. In practice, the optimal filter requires knowledge or estimation of the signal and noise spectra. A simple estimation method consists in measuring the noise spectrum and computing S(ω) by subtracting the noise spectrum from the noisy signal spectrum. However, due to random variations of noise, spectral subtraction can result in negative estimates of the short-time magnitude or power spectrum. Different methods for reducing and removing the distortions due to the rectification process are proposed in [33].

Under the hypothesis that the noises have the same spectrum for each sensors, the Gauss-Markov estimator coefficients, for the case of three sensors array (regardless of what filter is used for the GCC procedures), are given by:

(15) $$\left[\begin{array}{c} d_{2GM}\\ d_{3GM} \end{array}\right]=\frac{2}{3}\left[\begin{array}{cc} 1\;1/2 & -1/2\\ 1/2\;1 & 1/2 \end{array}\right]\left[\begin{array}{c} d_{12GCC}\\ d_{13GCC}\\ d_{23GCC} \end{array}\right]$$

where $d_{ijGCC}$ are the DToAs between sensor *i* and *j* estimated by using the GCC procedure, whereas $d_{iGM}$ are the time delays with respect to the reference sensor estimated with the GM estimator. The weights of the Gauss-Markov estimator have a more complex form (expressed by a ratio of time delays variances) only when the noise spectra for each sensor, $N_{i}$, are not all equal.

In this paper, for testing the DoA estimation performance with the two clusters of Figure 1, we assumed equal white noise spectra and a flat signal spectrum within a Band $B_{s}$, to emulate the narrow-band impulsive signals due to an impact. In this case, the Optimum Filter (14) is equal to an arbitrary constant within the Band $B_{s}$, and 0 elsewhere:

(16) $${\left|F_{OPT_{Flat}}\left(\omega \right)\right|}^{2}=\left\{\begin{array}{c} 1\;\omega \in B_{s}\\ 0\;\mathrm{elsewhere} \end{array}\right.$$

The Band $B_{s}$, is estimated by using the spectral subtraction technique, assuming to know the white noise level. The distortions induced by the rectification of negative values of the estimated power spectrum *S* are neglected. This assumption is justified when SNR values are sufficiently high, while the non-linear distortions are not negligible when the signal-to-noise ratio decreases.

Finally, an optimal DoA estimation function from the estimated time delays has to be found to attain the CRB${}_{u-v}$ (the inverse of (9)). Given the designed array geometry (DC in Figure 1b), the following result is obtained:

(17) $$d_{2}=\frac{d}{v}2\cos\left(\alpha \right)\cos\left(\theta \right),\;d_{3}=\frac{d}{v}\left(\cos(\alpha )\cos(\theta )-(1+\sin(\alpha ))\sin(\theta )\right)$$

θ can be computed by using the ratio $\eta \overset{.}{\;=}d_{2}/d_{3}$ and inverting with respect to θ. The “true” relation between θ and η can be used as estimation function:

(18) $${\hat{\theta }}_{DC}=\mathrm{atan}\left(\frac{\tan\beta (\eta -2)}{\eta }\right),\;\mathrm{with}\;\beta =23{}^{\circ}$$

Note that the estimator ${\hat{\theta }}_{DC}$ is a function only the ratio $d_{2}/d_{3}$. From the Theory of Uncertainty Propagation [34], the mean square error and variance (when the time delays $d_{2},d_{3}$ are random variables with covariance matrix (4)) can be computed by expanding the estimation function in the first order Taylor series:

(19) $$e_{ms}=\sigma_{{\hat{\theta }}_{DC}}^{2}=E[\hat{\theta }]-\theta =\left[{\left(\frac{\partial f}{\partial d_{2}}\right)}^{2}+{\left(\frac{\partial f}{\partial d_{3}}\right)}^{2}+\left(\frac{\partial f}{\partial d_{2}}\right)\left(\frac{\partial f}{\partial d_{3}}\right)\right]\sigma_{d_{i}}^{2}$$

this formula is valid around the point of expansion $(E\left[d_{2}\right],E\left[d_{3}\right])$ of function *f* in Taylor series. Expanding Equation (19), we have:

(20) $$\begin{array}{c} \sigma_{{\hat{\theta }}_{DC}}^{2}={\left(\frac{\partial f}{\partial \eta }\right)}^{2}\left[\frac{d_{2}^{2}+d_{3}^{2}-d_{2}d_{3}}{d_{3}^{4}}\right]\sigma_{d_{i}}^{2}\\ =\frac{d_{2}^{2}\left(\theta \right)+d_{3}^{2}\left(\theta \right)-d_{2}\left(\theta \right)d_{3}\left(\theta \right)}{{\left({d_{2}}^{\prime }\left(\theta \right)d_{3}\left(\theta \right)-d_{2}\left(\theta \right){d_{3}}^{\prime }\left(\theta \right)\right)}^{2}}\sigma_{d_{i}}^{2} \end{array}$$

where the last equivalence is provided by the inverse function theorem. The function in (20) is precisely the integrand of (13), i.e., $I_{22}/\det(\mathrm{FIM})$, expressed in terms of DToAs $d_{i}\left(\theta \right)$, instead of elements $r_{i}\left(\theta \right)$, namely the Differences in Distance of Arrival (DDoAs).

## 5. Numerical Performance Comparison between Clusters and DoAs Estimators

In order to validate the design procedure of the array geometry and the performance of the proposed DoA estimator, a numerical analysis was performed. The estimation functions of the two clusters of Figure 1 are respectively:

(21) $${\hat{\theta }}_{SC}=\mathrm{atan}\left(\frac{d_{2}}{d_{3}}\right),\;{\hat{\theta }}_{DC}=\mathrm{atan}\left(\frac{\tan(23{}^{\circ})\left(d_{2}/d_{3}-2\right)}{d_{2}/d_{3}}\right)$$

In particular, impact waves propagating in aluminium plate 1 [mm] thick (Young’s modulus 70 [GPa], Poisson’s coefficient 0.3 and material density 2700 [kg/m${}^{3}$]) were simulated with the Greens function formalism adopted in [35]. The impulse response of a bandpass Batterworth filter (10th order) with different bandwidths and center frequencies was used in order to simulate the impact signal. The two DToAs with respect to the first sensor $d_{2},d_{3}$ were computed from the simulated acquired signals by using three different estimation modalities:

1. locating the peaks of the cross-correlation ([36]);
2. by using the Gauss-Markov estimator (15) consisting in three cross-correlation procedures;
3. by combining the Gauss-Markov estimator (15) with three GCC procedures (filtering first the signals with the filter (16);

The last modality is the optimum one in the considered case which involves (additive) white (zero-mean Gaussian) noise (AWGN) and quasi-flat signal spectrum *S* in a fixed band. The power spectrum S(ω) and its band $B_{S}$ for the filter (16) are estimated by the spectral subtraction technique.

Simulations were performed for multiple impact locations obtained by varying the true DoA with $5^{{}^{\circ}}$ steps (θ=−45°,−40°,−35°,…,45°), the distance from the cluster center being 0.8 [m]. The results achieved by the clusters of Figure 1 are given in Table 1, Table 2 and Table 3, for different center frequencies and bandwidths of the impact signal and different peak signal to noise ratios (PSNR). To assess the Standard Deviation (SD) of DoA estimations, 200 simulations, on the entire 90° sector, were performed. Furthermore, the maximum error (ME) over all simulations was considered. The simulations were run simulating the propagation of the A0 Lamb mode, and considering: (i) circular piezo sensors with radius equal to 5 [mm], (ii) the radius of the array *d* equal to 2 [cm], (iii) sampling frequency (Fs) equal to 2 [MHz].

As shown by the Table 1, Table 2 and Table 3, the SD and the ME values obtained with the designed cluster are, as expected, smaller w.r.t. the SC, for PSNR values higher than 24 dB. In particular, the best performances are achieved when the optimal DoA estimator, based on the GM-GCC time delays estimator, is used. In this case, the variances almost equate to the CRB${}_{u-v}$. Due to the wave dispersion of the A0 mode, the higher is the considered center frequency, the higher is the wave (group) velocity *v*. It is important to consider this fact because the DoA CRB in Equation (13) and the variance of the DoA estimator in Equation (20) increase as $v^{2}$. Conversely, for the case of quasi-flat signal spectrum in a given band $B_{S}$, the DToA variance term $\sigma_{d}^{2}$ of the Covariance Matrix (the CRMB in Equation (4) for an optimal estimator) decreases as $B_{S}^{3/4}+3B_{S}^{}\omega_{c}^{2}$, where $\omega_{c}$ is the central frequency of $B_{S}$ (see (5)). Therefore, the two terms, wave velocity and central frequency have an opposite influence on the DoA estimation performance (see the SD values of Table 1 and Table 2). Finally, it can be noted that the higher is the bandwidth, the smaller is the DToA and DoA variance (compare Table 1, Table 2 and Table 3 which are characterized by a 10 and 30 [kHz] Bandwidth, respectively).

## 6. Directive Base Sensor Design

In order to filter ASs with a DoAs out of considered angles range, a novel directive base sensor is investigated in this paper. The sensor beam pattern is ideally equal to 1 in a given range and 0 elsewhere. Without lack of generality, we refer to the [0°,90°] range as the one where the beampattern is equal to 1. The beampattern of a sensor is linked to its shape as described by the model proposed in [37] which estimates the frequency response of a piezo sensor impinged by a Lamb wave mode as:

(22) $$V_{P}\left(\omega \right)=jU\left(\omega \right)k_{0}\left(\omega \right)H_{P}\left(\theta \right)D_{P}\left(\omega ,\theta \right)$$

in this equation, U(ω) denotes the amplitude and the polarization of the wave component relevant to the piezo properties of the patch at the considered frequency, $k_{0}\left(\omega \right)$ is the wave vector that characterizes the propagation and $H_{P}\left(\theta \right)$ is a quantity related to the material properties of the piezo-structure system. Without lack of generality, here we consider the case of piezo patches with a single polarization and constant piezoelectric properties. Finally, the only function which depends by the DoA θ and by the piezo-sensor shape is the $D_{P}\left(\omega ,\theta \right)$ function. It defines the frequency response for all possible angles of arrival θ. Therefore, it is called Directivity function and can be computed by the following integral:

(23) $$D_{P}\left(\omega ,\theta \right)=\int_{\Omega_{P}}e^{-jk_{0}\left(\omega \right)\left(x\cos\theta +y\sin\theta \right)}\phi_{P}\left(x,y\right)dxdy$$

where $\phi_{P}\left(x,y\right)$ is the function that describes the geometry of sensor and is referred to as *shape function*. Defining $\Omega_{P}$ the area of the piezoelectric path, the shape function is equal to 1 when $\left(x,y\right)\in \Omega_{P}$ and 0 elsewhere.

Considering as an example a circular piezo-sensor of radius *R*, the (23) provides:

(24) $$D_{P}\left(\omega ,\theta \right)=2\pi R^{2}\frac{J_{1}\left(Rk_{0}\left(\omega \right)\right)}{Rk_{0}\left(\omega \right)}\approx 2\pi R^{2}\mathrm{sinc}\left(Rk_{0}\left(\omega \right)\right)$$

where $J_{1}\left(\cdot \right)$ is the first kind Bessel function of first order and $k_{0}\left(\omega \right)$ is the wave vector of the propagation mode of Lamb waves (e.g., A0 or S0 mode). Observe that the (24) doesn’t depend by θ but only by frequency, so the directive properties of a piezo-disk are the same for all angles, i.e., the disk is omnidirectional. We define the base sensor beampattern at frequency ω as;

(25) $$d\left(\omega ,\theta \right)=\frac{\left|D_{P}\left(\omega ,\theta \right)\right|}{\max_{\theta }\left[\left|D_{P}\left(\omega ,\theta \right)\right|\right]}$$

**Figure 2:**
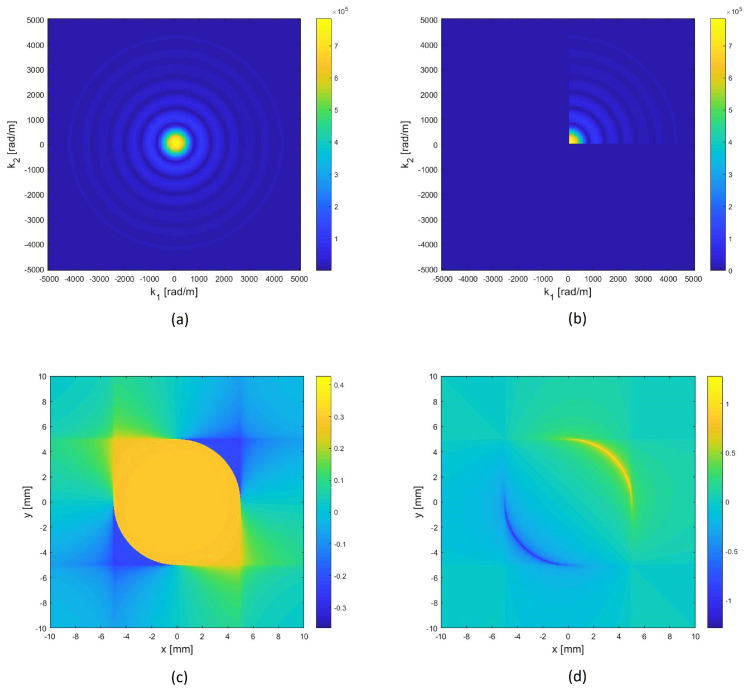
(**a**) Disk Directivity in k−space (|2D−FT|) (5.0 [mm] radius). (**b**) Imposed Directivity equal to that of a disk in [0°,90°] and 0 elsewhere. (**c**,**d**) Real part and imaginary part of 2D−IFT: the ideal shape functions continuously modulated.

For a circular sensor then d(ω,θ)=1. Thus, the Directivity function $D_{P}\left(\omega ,\theta \right)$ (23) is equal to the bi-dimensional spatial Fourier Transform (2D-FT) at angle θ of the shape function. Then, the shape function which corresponds to a given desired directivity can be determined with an Inverse Fourier Transform. In our approach, we impose the same Directivity function (and so the same frequency response) of a piezo-disk of given radius in the [0–90]° angles-sector and 0 elsewhere. Therefore, we compute the 2D-FT of a disk, set to 0 all values of 2D-FT in the k-space domain between [90;360]°, and finally get back in the space-domain, via the 2D-Inverse FT (IFT) to obtain the desired shape function (see Figure 2).

Note that a real shape function is achieved if and only if the Directivity function is symmetric with respect to the origin. Instead, our procedure tolerates the generation of a complex shape function, with a real part and an imaginary part, both having positive and negative values (Figure 2c,d). This higher complexity allows us to have a beam pattern that is not symmetrical, i.e., without lobes in the [180:270]° range. Unfortunately, the described procedure produces a continuously modulated shape function that cannot be realized in practice. So, a quantization procedure is applied to the computed shape function. In particular, the phase of the complex shape function is quantized as detailed in the Table 4.

**Figure 3:**
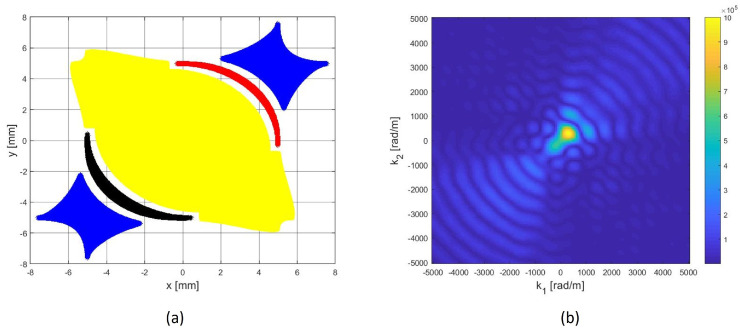
Directive Complex Sensor (DCS) designed to priviledge the [0°–90°] angular sector (**a**). The geometry of the piezopatches is generated by a quaternary phase quantization and a subsequent binary amplitude quantization of the continuously modulated shape functions illustrated in Figure 2. (**b**) 2D-FT of the complex quantized shape function: due to quantization procedure, it is not perfectly matched to the desired one depicted in Figure 2b but it is clearly asymmetrical.

Then, the absolute values greater than a certain positive threshold are set to 1 and others to 0 (see Figure 3). Areas associated with the same quantized values define the shape of the electrodes of the piezo patches used as sensors. A distance gap of at least 0.5 [mm] between the patches has been imposed to be compliant with the typical geometrical limitations that are associated with patch manufacturing.

Such a procedure generates the Directive Complex Sensor (DCS). As can be seen in Figure 3a, such a sensor consists of five piezo patches each one depicted with a different color. It is worth noting that the two blue patches related to the quantized phase π can be short-circuited. Moreover, the three patches corresponding to the opposite phases 0 and π (referred to as the *real part*) correspond to regions where the computed shape function has almost equal absolute average value. The same applies for the two patches related to phases π/2 and 3/2π (the *imaginary part*). This implies that piezo-patches related to the real part require just one differential acquisition channel and a second differential channel is required by the patches related to the imaginary part. In order to generate the (complex) time-signal, a weighted sum of the two acquired differential signals has to be performed, in which the signal related to the imaginary part is multiplied by the factor $i\cdot W_{Im}$, where *i* is the imaginary unit and $W_{Im}$ is a suitable weight. Finally, the anti-analytic signal of the complex acquired signal is computed and used to feed the DToA estimator.

Figure 3b shows the actual 2D-FT (absolute value) in the k-space domain, i.e., the 2D-FT computed after the quantization of the shape function. Due to quantization, the values of the 2D-FT, out of [0°,90°], are not 0, but are still smaller than values in the monitored angular sector.

**Figure 4:**
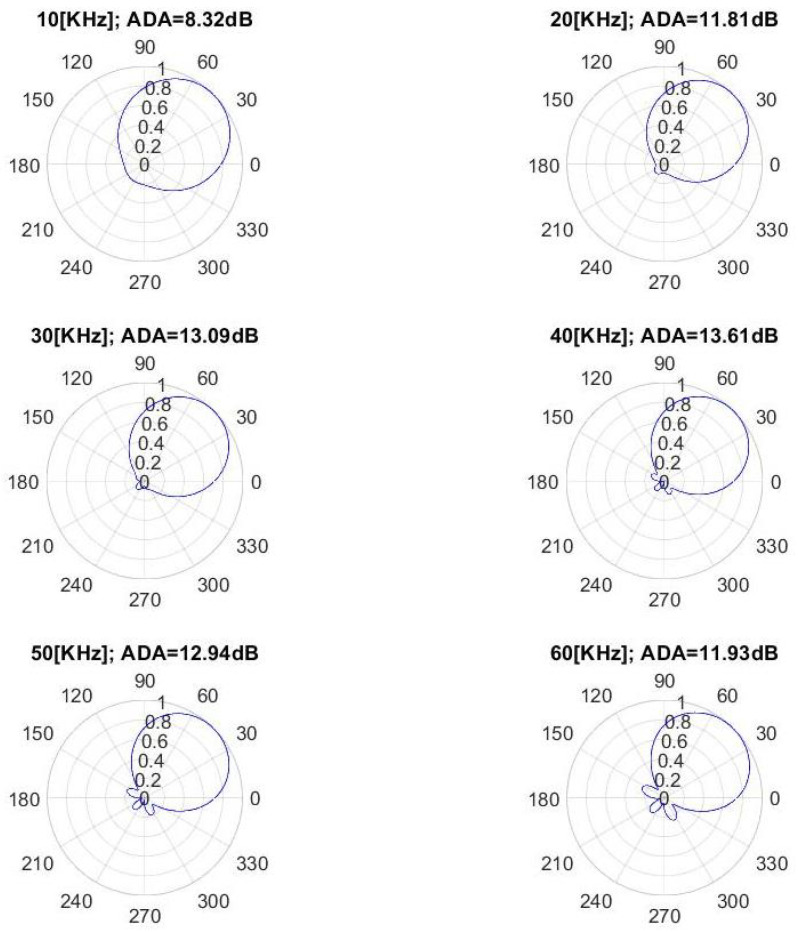
The DCS beampatterns computed at 6 different values of frequency when A0 mode propagating an aluminium plate (1 [mm] thick) and corresponding Average Directional Attenuation (ADA) values.

By using Equation (25), the DCS sensor theoretical beampatterns were computed at different values of frequency, considering the wave vector values of A0 mode when propagating in an aluminum plate with 1 [mm] thickness (i.e., for a known dispersion curve $k_{0}\left(\omega \right)$). As shown in Figure 4, useful directive beampatterns are achieved in the [10–60] [kHz] frequencies band. However, the best directional behavior is achieved in the [30–60] [kHz] frequency range.

The directivity properties at each frequency can be expressed by the Average Directional Attenuation (ADA) parameter. It is computed by the beampatterns values, as the ratio between the average beampattern value in the monitored angular sector (i.e., [0–90]°) and out of that one. In Figure 4, the ADA values for different frequency values are shown. The ADA value is above 8.3 dB in the [10–60] [kHz] band, and 13.0 dB in the [30–60] [kHz] band.

Regarding the $W_{Im}$ parameter, in order to find an optimal value a suitable cost function JWIm was defined:

(26) JWImW=1N∑i=1Nς(W,ki)DAαβ(W,ki)

where $k_{i}$ are N wave-numbers in the considered (spatial) spectral region, $DA_{\varphi }^{\gamma }\left(W,k_{i}\right)$ is the directional attenuation at angle φ w.r.t the one at angle γ and $\varsigma (W,k_{i})$ is equal to highest sidelobe level of beampatterns at each frequency $k_{i}$. By minimizing JWIm for $k_{i}=353,409,459,505\;\left[rad/m\right]$ (corresponding to the frequencies $f_{i}=30,40,50,60\;\left[kHz\right]$ of the A0 mode in the considered setup plate), φ = 120° and γ = 90°, the value WIm−Opt=6.81 is obtained. It is worth noting that the DA of the proposed DCS is sufficiently high to mask directional interferences in a given angular range $\left[\psi_{1},\psi_{2}\right]$, subset of [90°,360°]. For example, the DA is above 11.3 dB within [30–60] [kHz], for all undesired DoAs within [130–320] deg. This is due to DCS beampattern non-idealities, in particular to the non-sharp mainlobe cut-off near 0° and 90°. The DCS DA is also limited by the highest sidelobe level. The previous facts justified the cost function (26).

Such non-idealities can be attributed to the detrimental effect of binary quantization. More specifically, the DCS shows the better directivity properties in the wave vector *k* values range [353–505] [rad/m] (corresponding to the 30–60 [kHz] beampatterns shown in Figure 4). It is worth noting that the relationship between wave vector function and frequency $k_{0}\left(\omega \right)$ depends on the monitored structure characteristics (material, thickness, etc.). In other words, the optimal frequency range of the DCS can be found by taking into account the dispersion curves of the monitored medium.

## 7. Numerical Results of the Directive Complex Sensor

**Figure 5:**
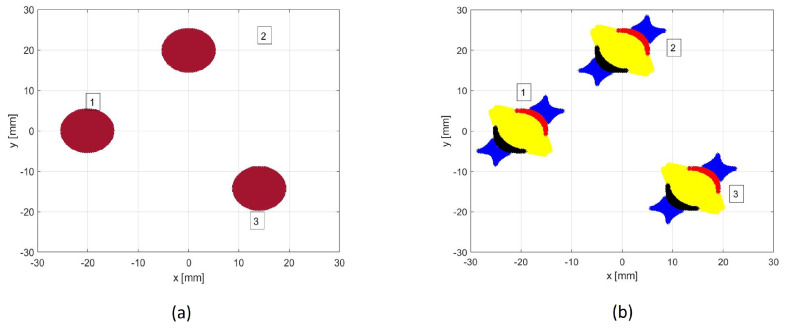
The Designed Clusters (DC) of Disk sensors (**a**) and of DCSensors (**b**) (rotated by 45º compared to Figure 1b) for an optimal DoA estimation in [0–90]°.

In order to validate the design procedure and the directivity properties of the DCS, in the following subsections, the beampatterns obtained from the Finite Element Method (FEM) are shown and compared with the theoretical ones (see Figure 4). Furthermore, a numerical comparison between the performance of DC (rotated by 45° to work within [0–90]°) of conventional disk-sensors and the same cluster with DCS (see Figure 5) is given, considering undesired AS with DoAs out of [0–90]°.

### 7.1. Finite Element Simulation Using COMSOL Multiphysics

**Figure 6:**
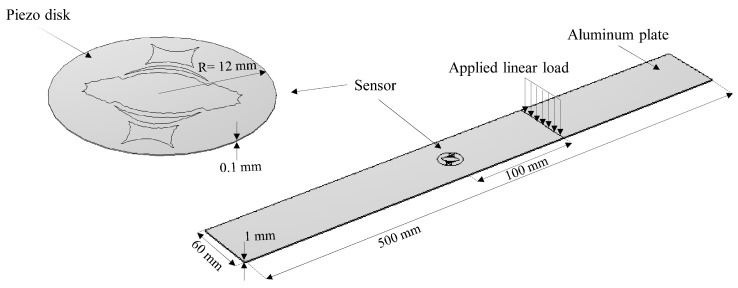
Three-dimensional geometry of the model.

To validate the DSC performance, the theoretical beampatterns predicted by the model have been compared with the ones resulting from finite element (FE) simulations. To do so, a three-dimensional COMSOL^®^ [38] based FE model of the proposed DSC was built. In the numerical model, an aluminum plate with dimensions of 500 [mm] × 60 [mm] and thickness 1 [mm] was chosen as the propagation medium. Since it is sufficient to shape just one metalization of the DCS (top or bottom) to achieve the desired directive behavior, the DCS was modeled using the geometry obtained in the design procedure, below which a small disk of piezoelectric material with a radius of 12 [mm] was defined. Then, the DCS was attached to the plate as shown in Figure 6.

**Figure 7:**
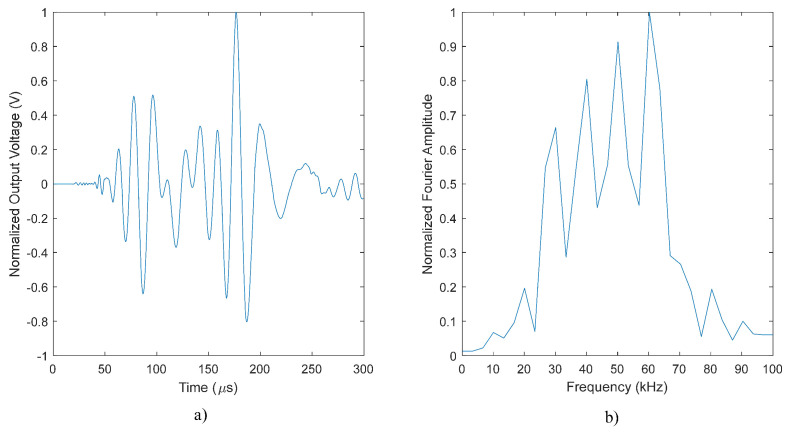
Sensor response for θ=0: (**a**) time plot and (**b**) frequency spectrum.

**Figure 8:**
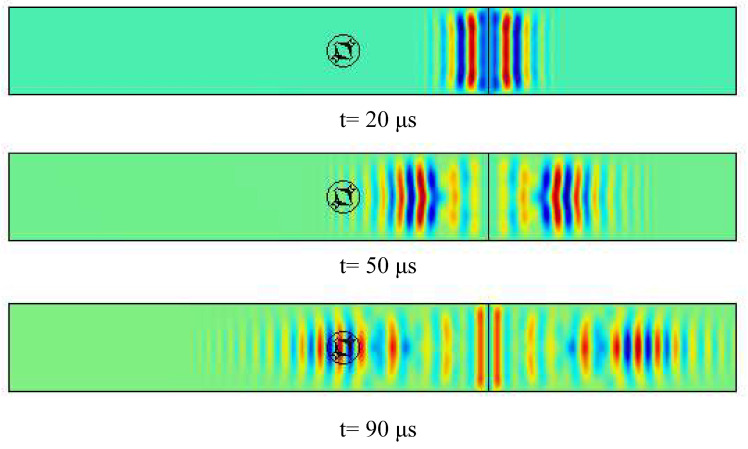
The generated wave field at different times for θ=0.

**Figure 9:**
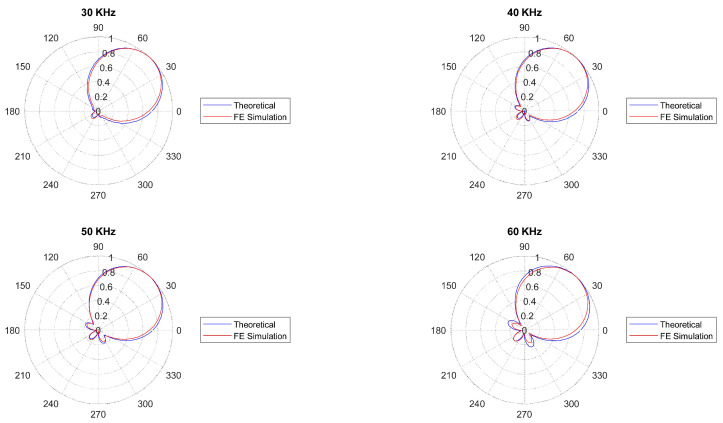
Comparison of the FE simulation and theoretical beampatterns computed at 4 different frequencies.

The excitation for the A0 mode was simulated using a line load in a way that a plane wave is generated within the plate. It should be noted that the excitation signal is considered as a sine-wave with a combination of four different frequencies of 30, 40, 50 and 60 kHz. Unlike the common procedure to compute the directivity pattern, which includes a number of point sources around a fixed transducer, a different approach was utilized here: at each simulation run, the sensor was rotated of 5 degrees, while the excitation load was fixed, as depicted in Figure 6. The motivation of using such a method was to reduce the computational cost of the numerical model. Furthermore, in order to prevent wave back-reflection at side boundaries, the Low-Reflecting Boundary option was utilized. Two physics, Structural Mechanics and Electrostatics were coupled by the Multiphysics-Piezoelectric Effect to take the solid mechanics of the aluminum plate and the electrical feature of the piezoelectric sensor into consideration. The simulation results including the sensor response and the generated wavefield at different times for θ=0 are given in Figure 7 and Figure 8, respectively. The beam patterns obtained from the FE simulation are compared to that of the theoretical model in Figure 9. A notably good agreement between them is observed, indicating the effectiveness of the proposed complex sensor.

### 7.2. DoA Estimation with DCS Clusters in Reverberant Environments

**Figure 10:**
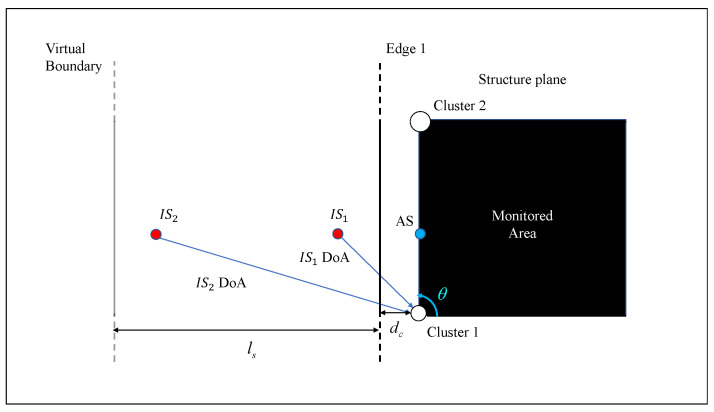
Example of directional interference due to edges reflections. $IS_{1}$ represents a coherent interference due to the edge reflection of the AS to be detected, whereas $IS_{2}$ represents an incoherent interference due to another acoustic source.

As discussed in the Introduction, in realistic reverberant environments, coherent reflections and incoherent reflections may hamper the DoA estimation. This interference can be viewed as waves generated by virtual *Image Sources (ISs)*, due to the mirroring produced by the boundaries of the monitored structure [39]. In the most general case, undesired AE is given by multiple ISs. Let us suppose that the AS signal is corrupted by undesired reflections due to an edge closely spaced w.r.t. the sensor cluster, as shown in Figure 10. In this example, the AS is placed in the position specified by the blue circle (DoA equal to 90°), while Edge 1 generates an IS ($IS_{1}$) which, in turn, generates coherent interference on the signal acquired by the DCS cluster.

Additional incoherent interference may be generated by ISs of previous acoustic events. Both coherent and incoherent directional interference could have a detrimental effect on DoA estimation. Improved DoA estimation performance is achieved when the DoAs of ISs occur in angular ranges $\left[\psi_{1},\psi_{2}\right]$ filtered by the beampattern of the DCS, which ensures a minimum DA level.

In order to evaluate the DoA estimation performance of DCS clusters in realistic simulation setups, the cases of coherent and incoherent reflection interferences are considered. For both cases, in the [0–90]° angular-sector, the wave to be detected were simulated by changing their orientation with a step of 5°.

At first, coherent interference was simulated, the cluster was placed at $d_{c}$ = 17 [cm] from the edge, whereas the AS location distance was set equal to 40 [cm]. The directional interference $IS_{1}$ is produced by the mirroring of the AS induced by edge reflections (see the Figure 10: the AS DoA is equal to 90°, whereas the corresponding $IS_{1}$ DoA is equal to 130.36°). Considering a sampling frequency $f_{s}$ equal to 2 [MHz], a 200 samples Tukey window (i.e., a rectangular window with the first and last 47.5 percent of the samples equal to parts of a cosine) filtered by using a Butterworth filter (10th order) with a bandpass equal to [30–40] [kHz] was used as impact signal and as IS.

Considering the Design Array configuration depicted in Figure 5, the DoAs estimated by the processing of the simulate response (via the GM-GCC time delays estimator) of piezo disk-sensors and DCSs, together the actual AS DoA and the corresponding IS DoA for 19 different simulated angles cases are reported in Table 5. In these conditions, the Standard Deviation and the Maximum Error values are equal to 18.48 deg and 70.76 deg for the piezo-disk cluster, and 1.47 deg and 3.33 deg for the DCSs cluster, respectively.

**Figure 11:**
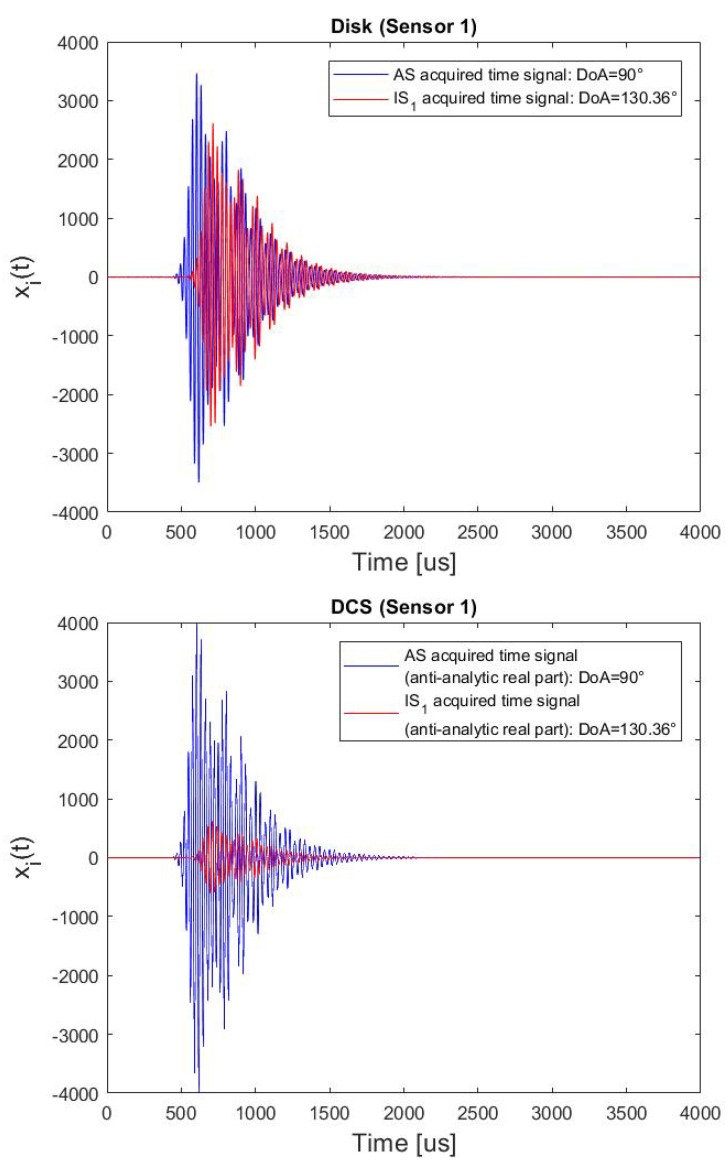
Superposition of two acquired signals: the AS to be detected, and the coherent edge-reflection due to an IS, when (top plot) the sensor is a Disk, and (bottom plot) a DCS. Impact distance *d* = 40 [cm]; Cluster distance from the edge $d_{c}$ = 17 [cm].

Two examples of acquired time signals, distinguishing the signal component related to the wave to be detected and the reflection, on a piezo Disk and on a DCS for the same DoAs are illustrated in Figure 11 (more specifically, for the DCS, the anti-analytic real part of the complex time signal is plotted). As can be seen, the AS signal and the IS signal are overlapped in time, hence a very unfavorable condition for DoA estimation. However, the DCS clearly shows the capability to strongly attenuate the spurious component.

In order to assess the DoA estimation performance in even more challenging conditions, the case of measurements affected both by directional interference and diffuse noise (AWGN) was considered. 200 simulations of AWGN, on the entire 90° sector, were performed for different PSNR values. The Standard Deviation and the Maximum values are given in Table 6.

Then, we considered the case of an additional incoherent interference with random DoA in the angular-sector ([169.69–180]°) (to simulate an undesired incoherent component due to an $IS_{2}$ of a previous impact/defect).

In particular, we have simulated AS and incoherent spurious waves impinging on the sensors simultaneously, because these are the most critical conditions to perform the DoA estimation. The impulse response of a [30–40] [kHz] band Chebyshev Type I filter (10th order with a passband ripple of 4 dB) with a 3 dB amplification (to simulate a high energy impact) was used as actuating signal of the incoherent component.

**Figure 12:**
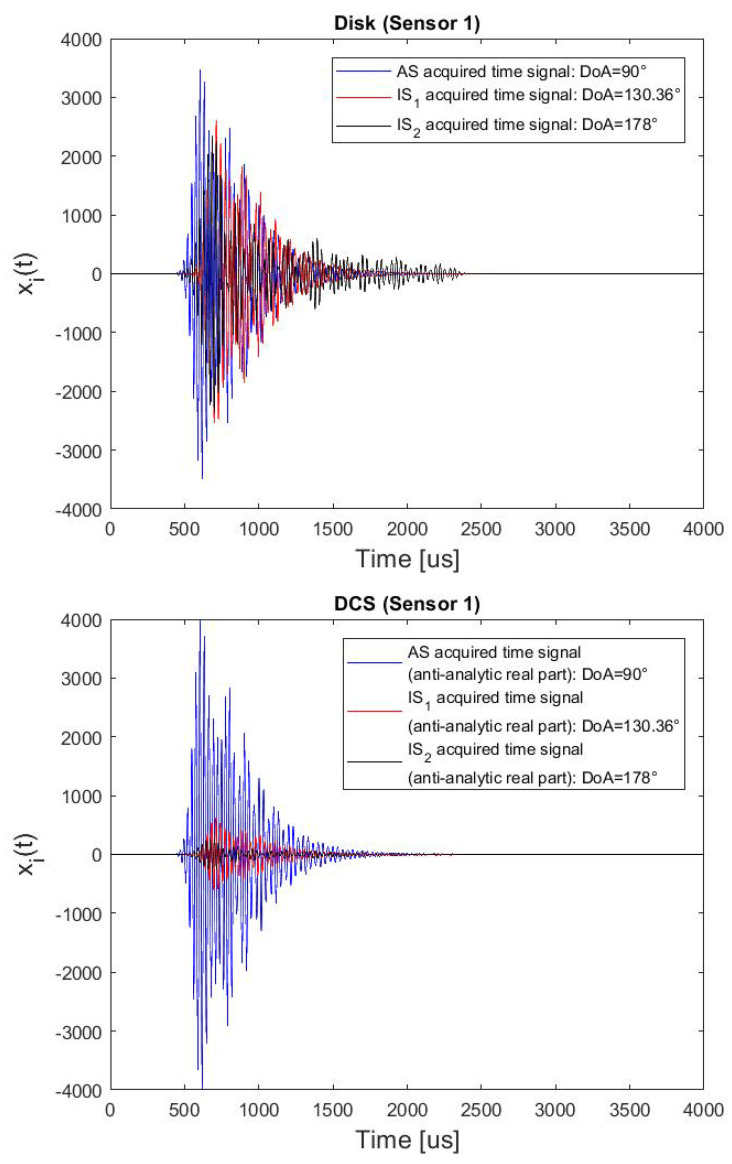
Superposition of three acquired signals, the AS to be detected and to two undesired components (coherent and incoherent interference due to two ISs of the current AS and the AS of a previous impact/defect), when the sensor is a Disk (top plot) and a DCS (bottom plot).

The DoA estimation results in the presence both of a coherent and incoherent interference by means of the cluster of disk sensors and DCSs (Figure 5), for 19 different simulated cases are reported in Table 7. The Standard Deviation and the Maximum Error values are equal to 31.4 deg and 67.3 deg for the piezo-disk cluster, and 2.03 deg and 5.22 deg for the DCSs cluster, respectively. Three examples of acquired time-signals, distinguishing the signal component related to the wave to be detected and the spurious ones, on a piezo Disk and on a DCS for the same DoAs are given in Figure 12. Also, in this case, the spurious components are strongly attenuated by the directional sensor.

Finally, the DoA estimation performance was evaluated when the measurements are affected also by diffuse noise (AWGN). 200 simulations of AWGN were performed for different PSNR values. The Standard Deviation and the Maximum values, shown in Table 8, clearly validate the ability of the DCS to cancel out *multiple spurious interferences*.

It is worth noting that the analyzed configuration is representative of many realistic scenarios. In all the considered cases, there is a clear advantage in the performance achieved by the DCS cluster with respect to the conventional piezo disks. The performance of the DCS is, however, degraded when the ISs and the AS to be detected are generated at closely spaced locations. This is due to the fact that the directional selectivity of the DCS is not perfect, and, in case of interferences whose direction of arrival is slightly larger than 90° or slightly less than 0°, the attenuation is poor.

It is also worth noting that the proposed DCS cluster provides good results in DoA estimation both for coherent and incoherent directional interference even for low PSNR values because the DoA estimator (21), based on the GM-GCC estimator, is the optimal estimator in presence of noise, for any SNR value. Viceversa, the GCC-PHAT signal processing is useful just for high SNR values because is based uniquely on the signal phase information. Furthermore, the DCS sensors allow to work on all signal time-lapse, whereas the commonly-adopted selection of smaller time windows reduces the DoA estimation accuracy, in particular when the non-impulsive and long-lasting signal is to be detected.

## 8. Conclusions

In this work, an optimal N-sensor array design procedure for DoA estimation is discussed. Specifically, the minimization of the CRB of the DoA with a Bayesian approach is used as an optimality criterion, considering the wave velocity to be *unknown*.

The general procedure was applied to design a 3-sensor cluster to monitor a 90° sector. Two of such clusters can be used to detect and locate Acoustic Sources such as crack growth emissions or impacts over a rectangular area.

An efficient DoA estimator was found, based on the GCC-Gauss Markov estimator of the DToAs. It was shown that the optimal GCC is a band-pass filter, for the case of white noise and narrow-band quasi-flat signal spectrum whose band can be estimated via the subtraction of noise spectrum. The Designed Cluster and DoA estimator were validated by numerical simulations.

Despite the good performances achieved, it must be considered that, in realistic scenarios, many physical sources can generate directional interference. This is the case of waves due to reverberation, for example. To filter out this interference, a novel directive passive sensor was designed to replace the piezo-disks which are conventionally adopted in this application field. The novel sensor exploits its shape as a means to attenuate spurious waves coming from directions that are not included in the monitored angular sector. It consists of five-piezo patches whose output is collected by two differential channels. The new sensor is able to filter directional interference in a known *k* wave vector bandwidth ([353–505] [rad/m]). In this band, the average attenuation for spurious waves is 12.89 dB. Future developments are aimed to improve the quantization procedures to achieve even better directivity in a larger bandwidth and to provide experimental results of the proposed DCS concept in different practical scenarios.

## Figures and Tables

**Table 1:** Comparison of Standard Deviation (SD) and Maximum Error (ME) values (in [Deg]) between the arrays of Figure 1 for noise-affected measurements at different PSNR values. Actuated pulse band: [30–40] [kHz].

	Standard Cluster						Designed Cluster					
	CC		GM-CC		GM-GCC		CC		GM-CC		GM-GCC	
PSNR	SD	ME	SD	ME	SD	ME	SD	ME	SD	ME	SD	ME
60 dB	0.45	1.05	0.44	1.05	**0.43**	**1.05**	0.35	0.67	0.29	0.63	**0.29**	**0.63**
40 dB	0.94	3.14	0.80	3.30	**0.46**	**1.86**	0.84	2.94	0.59	1.84	**0.32**	**1.16**
35 dB	1.53	4.99	1.27	5.05	**0.52**	**1.86**	1.46	6.1	0.98	3.6	**0.37**	**1.19**
30 dB	2.50	8.13	2.14	7.50	**0.67**	**2.58**	2.35	8.8	1.62	5.82	**0.51**	**1.92**
28 dB	3.09	10.10	2.55	10.19	**0.79**	**3.48**	2.86	11.09	1.97	7.73	**0.59**	**2.25**
27 dB	3.50	12.41	2.90	12.46	**1.80**	**8.31**	3.24	10.82	2.25	7.79	**1.73**	**6.02**
26 dB	3.85	12.30	3.18	11.20	**2.74**	**10.47**	3.68	12.78	2.52	9.45	**2.09**	**7.88**
24 dB	4.78	15.30	3.98	14.44	**3.64**	**12.85**	4.57	20.67	3.1	11.17	**2.83**	**9.87**

**Table 2:** Comparison of Standard Deviation (SD) and Maximum Error (ME) values (in degrees) between the arrays of Figure 1 for noise-affected measurements at different PSNR values. Actuated pulse band: [50–60] [kHz].

	Standard Cluster						Designed Cluster					
	CC		GM-CC		GM-GCC		CC		GM-CC		GM-GCC	
PSNR	SD	ME	SD	ME	SD	ME	SD	ME	SD	ME	SD	ME
60 dB	0.58	1.05	0.52	1.05	**0.52**	**1.05**	0.44	0.92	0.28	0.83	**0.26**	**0.83**
40 dB	1.22	4.09	1.02	4.03	**0.55**	**1.97**	1.1	4.31	0.77	2.49	**0.37**	**1.26**
35 dB	1.93	6.99	1.61	6.95	**0.63**	**2.91**	1.83	8.7	1.26	4.31	**0.44**	**2.06**
30 dB	3.31	11.22	2.72	11.05	**0.84**	**3.03**	3.03	13.41	2.11	8.58	**0.62**	**2.31**
28 dB	4.14	14.2	3.41	13.05	**1**	**4.88**	3.76	14.42	2.58	9.6	**0.93**	**3.51**
27 dB	4.52	16.04	3.72	14.96	**2.01**	**8.77**	4.26	15.55	2.93	12.2	**2.52**	**9.18**
26 dB	5.08	16.14	4.23	15.77	**3.92**	**15.97**	4.79	16.89	3.23	12.34	**2.86**	**9.79**
24 dB	6.36	21.69	5.28	20.28	**4.86**	**20.9**	5.84	20.7	4.01	15.1	**3.78**	**12.78**

**Table 3:** Comparison of Standard Deviation (SD) and Maximum Error (ME) values (in degrees) between the arrays of Figure 1 for noise-affected measurements at different PSNR values. Actuated pulse band: [30–60] [kHz].

	Standard Cluster						Designed Cluster					
	CC		GM-CC		GM-GCC		CC		GM-CC		GM-GCC	
PSNR	SD	ME	SD	ME	SD	ME	SD	ME	SD	ME	SD	ME
60 dB	0.35	0.85	0.34	0.85	**0.34**	**0.85**	0.45	0.72	0.4	0.72	**0.4**	**0.72**
40 dB	0.48	1.87	0.45	1.87	**0.37**	**1.42**	0.46	1.62	0.37	1.08	**0.38**	**0.72**
35 dB	0.73	2.28	0.64	2.3	**0.41**	**1.87**	0.63	2.31	0.46	1.76	**0.37**	**1.08**
30 dB	1.14	4.05	0.96	4.16	**0.48**	**1.87**	1.04	4.03	0.72	3.11	**0.38**	**1.08**
28 dB	1.35	5.1	1.15	4.83	**0.49**	**1.87**	1.25	4.69	0.87	3.15	**0.39**	**1.12**
27 dB	1.5	5.07	1.25	5.2	**0.54**	**2.14**	1.39	4.92	0.96	4.35	**0.51**	**2.08**
26 dB	1.67	5.53	1.4	5.46	**1.1**	**4.53**	1.54	6.08	1.07	3.75	**0.82**	**3.75**
24 dB	2.05	6.61	1.69	6.61	**1.59**	**6.21**	1.88	7.09	1.3	4.67	**1.2**	**4.57**

**Table 4:** Phase quantization scheme used in the complex shape function implementation.

Phase Interval	Quantized Value	Patch Color in Figure 3a
[−π/4+π/4)	0	Yellow
[π/4+3/4π)	π/2	Red
[3/4π+5/4π)	π	Blue
[5/4π+7/4π)	3/2π	Black

**Table 5:** Comparison of DoA estimation performance between the two arrays depicted in Figure 5 for measurements affected by a coherent edge reflection due to an Image Source (see Figure 10). In the table, the nominal DoA value, the direction of the interferring source, and the estimated values are reported in degrees (ASs band: [30–40] [kHz]; Impact distance d = 40 [cm]; Cluster distance from the edge $d_{c}$ = 17 [cm]).

		Disks	DCSs			Disks	DCSs
		GM-GCC	GM-GCC			GM-GCC	GM-GCC
AS DoA	IS DoA	Estimated DoA	Estimated DoA	AS DoA	IS DoA	Estimated DoA	Estimated DoA
**0**	180	2.71	**−0.93**	**50**	160.27	60.14	**49.85**
**5**	178.24	3.93	**3.93**	**55**	157.7	46.95	**56.98**
**10**	176.48	11.01	**9.19**	**60**	154.91	53.05	**58.91**
**15**	174.68	16.14	**14.94**	**65**	151.87	66.43	**66.18**
**20**	172.86	22.81	**19.58**	**70**	148.51	72.26	**73.16**
**25**	170.98	27.23	**25.52**	**75**	144.77	97.54	**74.07**
**30**	169.04	28.96	**30.34**	**80**	140.56	91.82	**82.38**
**35**	167.02	30.71	**36.31**	**85**	135.8	14.23	**83.89**
**40**	164.9	37.06	**40.15**	**90**	130.36	104.67	**93.33**
**45**	162.66	47.31	**45.95**	-	-	SD 18.48	SD 1.47
						ME 70.76	ME 3.33

**Table 6:** DoA estimation performance (SD and ME in degress) for the DCS cluster shown in Figure 5) when the measurementes are affected by a coherent edge reflection (simulation setup of Table 5) and diffuse noise (AWGN).

**PSNR**	60 dB	30 dB	20 dB	15 dB	10 dB	9 dB	8 dB	7 dB
**SD**	*1.5*	*1.52*	*1.65*	*1.9*	*2.47*	*2.63*	*2.85*	*3.11*
**ME**	*3.33*	*4.58*	*5.69*	*6.69*	*9.24*	*9.57*	*11.91*	*12.99*

**Table 7:** Comparison of DoA (in degrees) estimation performance between two designed arrays (Figure 5) for measurements affected by a coherent edge reflection due to an IS (see the $IS_{1}$ in the Figure 10), and a incoherent spurious signal due to a second IS of a previous impact/defect with a random DoA within the range [169–180] (see the $IS_{2}$ in the Figure 10). AS and ISs band: [30–40] [kHz]; (Impact distance d = 40 [cm]; Cluster distance from the edge $d_{c}$ = 17 [cm]; Structure length $l_{s}$ = 1.1 [m]).

			Disks	DCSs				Disks	DCSs
			GM-GCC	GM-GCC				GM-GCC	GM-GCC
AS DoA	IS DoA	IS-2 DoA	Estimated DoA	Estimated DoA	AS DoA	IS DoA	IS-2 DOA	Estimated DoA	Estimated DoA
**0**	180	178	16.66	**−2.03**	**50**	160.27	171	69.3	**49.85**
**5**	178.24	179	7.62	**5.42**	**55**	157.7	179	28.44	**58.49**
**10**	176.48	171	17.76	**10.07**	**60**	154.91	179	116.24	**58.91**
**15**	174.68	179	32.01	**15.4**	**65**	151.87	175	20.26	**68**
**20**	172.86	176	29.71	**20.23**	**70**	148.51	178	83.38	**73.16**
**25**	170.98	171	30.2	**25.38**	**75**	144.77	171	83.52	**73.62**
**30**	169.04	172	23.41	**28.9**	**80**	140.56	174	27.38	**81.15**
**35**	167.02	175	25.85	**34.42**	**85**	135.8	179	17.65	**79.78**
**40**	164.9	179	61.1	**40.15**	**90**	130.36	178	110.89	**92.33**
**45**	162.66	179	92.82	**46.87**	-	-		SD 31.49 ME 67.35	**SD 2.03** **ME 5.22**

**Table 8:** DoA estimation performance (SD and ME in degress) by means of a DCS designed cluster (Figure 5)) when the measurementes are affected by a coherent edge reflection (simulation setup of Table 5) and diffuse noise (AWGN).

**PSNR**	60 dB	30 dB	20 dB	15 dB	10 dB	9 dB	8 dB	7 dB
**SD**	*2.04*	*2.04*	*2.13*	*2.53*	*3.38*	*3.54*	*4.26*	*4.04*
**ME**	*5.22*	*5.67*	*7.14*	*9.28*	*13.15*	*16.07*	*16.04*	*18.05*

## Data Availability

Not applicable.

## Abbreviations

The following abbreviations are used in this manuscript:

ADA	Average Directional Attenuation
AE	Acoustic Emissions
AS	Acoustic Source
AWGB	Additive White Gaussian Noise
CC	Cross Correlation
CRB	Cramér Rao Bound
CRB${}_{u-v}$	Cramér Rao Bound for unknown velocity of propagation
CRMB	Cramér Rao Matrix Bound
DA	Directional Attenuation
DC	Designed Cluster
DCS	Directive Complex Sensor
DDoA	Difference in Distance of Arrival
DoA	Direction of Arrival
DToA	Difference in Time of Arrival
FEM	Finite Element Method
FI	Fisher Information
FIM	Fisher Information Matrix
Fs	Sampling Frequency
FT	Fourier Transform
GCC	Generalized Cross Correlation
GCC-PHAT	Generalized Cross Correlation with phase transform
GM	Gauss Markov
GW	Guided Waves
IFT	Inverse Fourier Transform
IS	Image Source
ME	Maximum Error
ML	Maximum Likelihood
MUSIC	Multiple Signal Classification
pdf	probability density function
PSNR	Peak Signal to Noise Ratio
SC	Standard Cluster
SD	Standard Deviation
SHM	Structural Health Monitoring
SNR	Signal to Noise Ratio
2D-FT	Bi-dimensional Fourier Transform
2D-IFT	Bi-dimensional Inverse Fourier Transform
